# A rare association of deformities with diplopodia, aplasia of the tibia and double fibula: A case report

**DOI:** 10.1186/1752-1947-2-102

**Published:** 2008-04-07

**Authors:** Shah Alam Khan, Ashok Kumar, Manish Kumar Varhney

**Affiliations:** 1 Department of Orthopaedics, All India Institute of Medical Sciences, Ansari Nagar, New Delhi-110029, India

## Abstract

**Introduction:**

The association of fibular duplication with metatarsal diplopodia is extremely rare with only a few cases reported in the medical literature.

**Case presentation:**

We present a 4-month-old girl with left tibial agenesis with fibular duplication (mirror foot) and metatarsal diplopodia.

**Conclusion:**

The case report highlights the need for an understanding of this rare congenital anomaly which may be seen only once in the working lifetime of an orthopaedic surgeon.

## Introduction

Diplopodia, which is an accessory tarsal or metatarsal bone with double fibula, is an extremely rare condition. It has to be differentiated from polydactyly, where accessory tarsal or metatarsal bones are not seen and is a relatively innocuous condition both in terms of diagnosis and management.

## Case presentation

**Figure 1:**
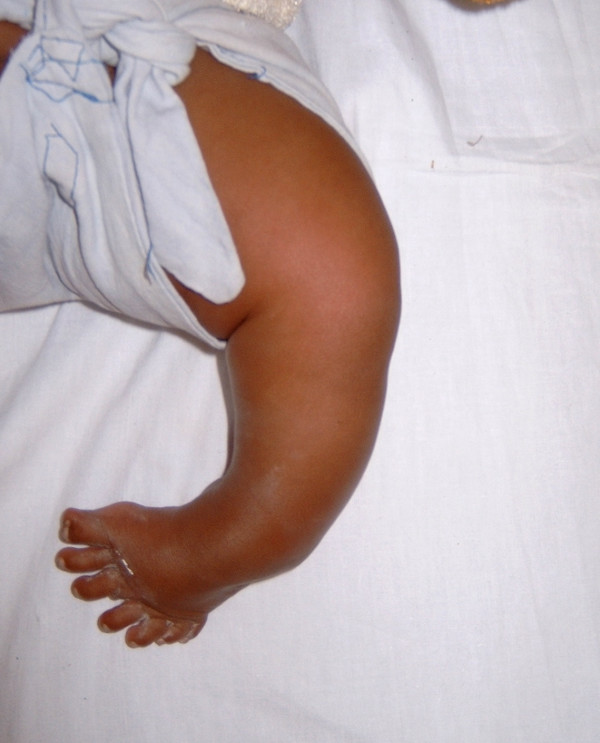
Clinical photograph of the left foot showing the seven toes and equinovarus deformity.

**Figure 2:**
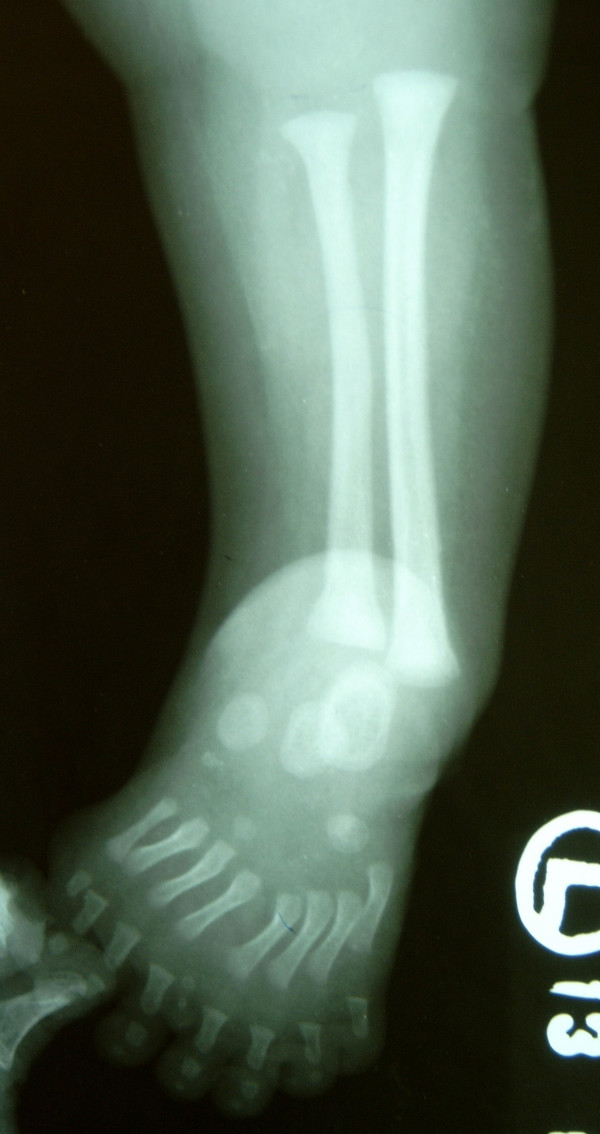
AP radiograph of the left lower limb with the foot showing two fibulae with multiple metatarsals.

**Figure 3:**
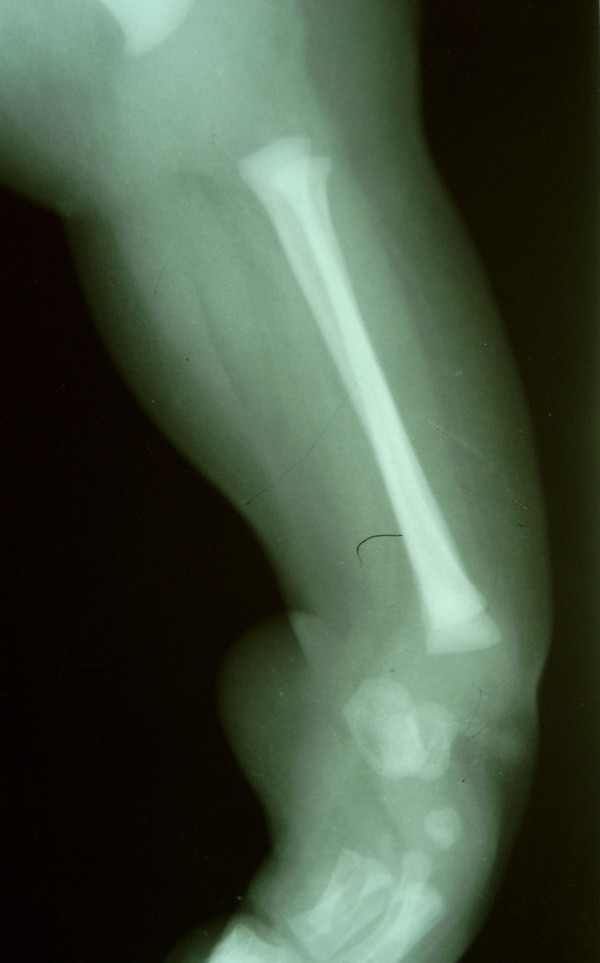
Lateral radiograph of the left leg showing anterior subluxation of the distal end of the tibia over the talus.

A four-month-old female child with deformity of her left leg and seven toes on her left foot was brought to the clinic by her parents. She was their first child and there was no obvious family history. The mother gave a non-contributory antenatal history. On examination, the baby had a short left leg with bony prominence laterally around the knee joint. Clinically, the normal bony contours of the knee joint were not palpable. Medially the normal contour of the tibia was not felt. The left foot was short, broad and had an equinovarus deformity and a total of seven toes (Fig. 1). General visceral examination was normal. Her remaining musculoskeletal examination, including that of the right lower limb, was normal. Anteroposterior radiograph (Fig 2) of the left lower limb showed two fibulae, with absence of the tibia, patella and the actual knee joint line. The medial fibula was shorter than the lateral fibula and its inferior end was seen articulating with the talus. The left foot had eight metatarsals with ossification centres for talus, calcaneum, cuboid and navicular. The cuboid was articulating with the 7^th ^and 8^th ^metatarsals. The great toe had two metatarsals articulating with one phalanx medially and three phalanges laterally. The third toe had two extra phalanges and the 7^th ^digit had only one phalanx. Lateral radiograph of the left leg (Fig 3) showed anterior subluxation of the distal end of the leg over the talus. A clinico-radiological diagnosis of tibial qgenesis with fibular duplication (mirror foot) and metatarsal diplopodia was made.

The equino-varus element of the foot deformity was corrected using serial casting. The child was kept under observation and the parents were advised about the possibility of a future above knee amputation being necessary.

Diplopodia (accessory tarsal or metatarsal bone) with double fibula is an extremely rare condition. It has to be differentiated from polydactyly, where accessory tarsal or metatarsal bones are not seen [1]. The condition is known to be associated with congenital heart anomalies, mainly atrial septal defect [2]. There was no congenital heart anomaly in our patient. Authors have reported a wide array of soft tissue anomalies along with the bony deformities in dissected specimens of these limbs [3,4]. Duplication of the triceps surae muscles and of the extensor hallucis muscle is common [3].

Treatment is controversial. Initial treatment is conservative with plaster application to correct the equinovarus at the ankle. Following plaster applications, surgical removal of the supernumerary foot should be undertaken followed by reconstruction of the ankle and knee joints. If the limb length discrepancy is extreme, or if the deformity at the ankle is grotesque, amputation can be performed to limit further disability and improve the quality of life of the child [3].

## Conclusion

Our case report highlights a rare congenital association between fibular duplication and metatarsal diplopodia. We feel that all babies with abnormal accessory toes should be evaluated for this particular anomaly and X-rays should be taken of the leg, rather than of the foot alone, to ensure a proper diagnosis.

## Competing interests

The author(s) declare that they have no competing interests.

## Authors' contributions

SAK identified the case and prepared the manuscript. AK and MKV helped in manuscript preparation. All the authors have read and approved the final version of the case report.

## Consent

We are thankful to the parents of our patient who kindly consented to allow us to publish this case report and any accompanying images. A copy of the written consent is available for review by the Editor-in-Chief of this journal.
